# Down-Regulation of Ribosomal S6 kinase RPS6KA6 in Acute Myeloid Leukemia Patients

**DOI:** 10.22074/cellj.2016.4310

**Published:** 2016-05-30

**Authors:** Mohammad Rafiee, Mohammad Reza Keramati, Hosein Ayatollahi, Mohammad Hadi Sadeghian, Mohieddin Barzegar, Ali Asgharzadeh, Mohsen Alinejad

**Affiliations:** 1 Hematology Lab, Imam Reza Hospital, Faculty of Medicine, Mashhad University of Medical Sciences, Mashhad, Iran; 2 Cancer Molecular Pathology Research Center, Ghaem Hospital, Faculty of Medicine, Mashhad University of Medical Sciences, Mashhad, Iran

**Keywords:** Extracellular Signal-Regulated Kinase, Mitogen Activated Protein Kinase, Ribosomal Protein S6 Kinase, Gene Expression, Acute Myeloid Leukemia

## Abstract

**Objective:**

Signaling pathways such as extracellular regulated kinase/mitogen activated protein kinase (ERK/MAPK) have increased activity in leukemia. Ribosomal 6 kinase (*RSK4*) is a factor downstream of the MAPK/ERK pathway and an important tumor suppressor which inhibits ERK trafficking. Decrease in *RSK4* expression has been reported in some malignancies, which leads to an increase in growth and proliferation and eventually poor prognosis. In this study we measured *RSK4* expression rate in acute myeloid leukemia (AML).

**Materials and Methods:**

This cross-sectional study was undertaken in 2013-2014 at Ghaem Hospital in Mashhad, Iran, on 40 AML patients and 10 non-AML patients as the control group. The expression rate was measured by real-time polymerase change reaction (PCR) and employing the ΔΔCT method. Data were analyzed using Mann-Whitney and Spearman tests using SPSS (version 11.5).

**Results:**

Expression rate of *RSK4* was significantly decreased in the AML group in comparison with the non-AML group (P<0.001). There was also a significant decrease in *RSK4* expression in AML with t(15;17) in comparison to other translocations (P=0.004).

**Conclusion:**

We detected a down-regulation of *RSK4* in AML patients. This may lead to an increase in the activity of the ERK/MPAK pathway and exacerbate leukemogenesis or the prognosis of the patients.

## Introduction

A proportion of acute myeloid leukemia (AML) cases exhibit irregularities in the expression of genes including transcription factors, oncogenes, tumor suppressors, and abnormal activities in tyrosine kinase receptors which regulate blood production (1). Tyrosine kinase receptors recognize a specific sequence of enzymes (kinases) to apply the effect of their ligand on transcription of the genes (2). Extracellular regulated kinase/mitogen activated protein kinase (ERK/MAPK) is one of the wellknown pathways in blood cells which become activated by growth factors and cytokines (such as IL-3), through tyrosine kinase receptors (3). After the ligand attaches to the receptor, the RAS oncogene is activated which in turn activates the ERK (4,5). The activation of this pathway increases the chance of survival, growth and proliferation of hematopoietic cells (6,7) and also inhibits many of the apoptosis-related proteins such as caspase 9 (8). Irregularity in ERK/MPAK adjustment is an important factor in leukemogenesis of AML cells (9,10). In one study, increased activity of this pathway was related to weak/faint prognosis (11). Activation of ERK was also observed in more than 50% of primary AML progenitors, and this activation is an independent prognostic factor for survival in AML(12). Resistance to drugs is the major problem in effectively treating AML(13). It has been observed that inhibition of the ERK/MPAK signaling pathway by lovastatin and PD98059 (ERK inhibitory drug), resulted in apoptosis and treatments were better (14). Therefore the activity of this pathway and its related mediators may be relevant therapeutic targets in AML progression prevention (15). 

An important mediator in this pathway is ribosomal 6 kinase (RSK) which is also the most important substrate of ERK (4). The RSK family has four members (RSK1-4) and are activated by ERK (16). In spite of other members increasing growth and proliferation in cells, *RSK4* (known as RPS6KA6) has the ability to inhibit the ERK signaling pathway (17) and is also involved in "P53 dependent proliferation arrest" which therefore acts as a tumor suppressor (18). Also it has been reported that contrary to the other RSKs, UO126 (RSKs inhibitor) is not able to inhibit *RSK4* (17). In addition, *RSK4* has an inhibitory role in embryo development (19) and has association with *c-MYC* (a cell cycle regulator) (20). When DNA is damaged, *RSK4* is able to phosphorylate and activate check point kinase one (CK1) and halt the cell cycle progression (21). In colon, kidney and endometrial carcinoma, *RSK4* down-regulation has been observed (22). In fibroblasts, this decrease permits cells to pass the senescence induced by oncogenes and stress, and cause their immortality (3). In breast cancer, *RSK4* up-regulation reduces and limits the aggression and metastatic activities of tumor cells (23). 

Given that previous studies have shown *RSK4* to act as a tumor suppressor and also ERK/MPAK pathway exhibiting increased activity in AML, we analyzed *RSK4* expression changes in blood and bone marrow samples of AML patients by quantitative teal-time polymerase change reaction (PCR). 

## Materials and Methods

This cross-sectional study was performed in the Cancer Molecular Pathology Research Center, Ghaem Hospital, Mashhad University of Medical Sciences, Mashhad, Iran, in 2013-2014. The local Ethical Committee approved the research protocol and written informed consents were taken from all participants. Peripheral blood and bone marrow samples of diagnosed AML patients [according to World Health Organization (WHO) criteria] and those of non-AML individuals (as the control group) were collected in anticoagulant-containing blood sampling tubes Ethylene Diamine Tetra Acetic acid-K2 (EDTA-K2). For all patients, complete blood count (CBC) was analyzed and peripheral blood and bone marrow smears were prepared and observed after staining. AML was diagnosed as existence of at least 20% blasts in peripheral blood or bone marrow smears that were positive for myeloperoxidase or Sudan Black B staining and myeloid markers CD33, CD13, CD117, CD64 and CD14. According to morphologic features of the smears, other staining including periodic acid schiff and none-specific esterase were also performed. In the next step, for AML patients only, among the recurrent cytogenetic abnormalities determined by the WHO classification, three translocations including t(15;17), t(8;21) and inv(16) were analyzed by PCR (ABI thermo cycler, Applied Biosystems, USA). AML Patients without these genetic abnormalities were considered as a different group defined as "other". In addition, morphologic subtypes of all AML patients were determined according to the FAB classification (M0 to M7). AML patients undergoing treatment or with recurrent leukemia, uncertain diagnosis and improper samples were excluded from the study. The control group included individuals that had no neoplastic disorders in their history or their peripheral blood and bone marrow samples. To determine *RSK4* expression, initially RNA was extracted from mononuclear cells of the bone marrow samples (Tri-pure reagents, Roche Diagnostic, Germany) and cDNA was synthesised (Revertaid^TM^, Fermentas, Germany). Then real-time qPCR was undertaken to quantitate the expression level of *RSK4* using a SYBR Green master mix kit (Pars Tous, Iran) on an ABI thermocycler (One Step, USA). Differential expression was analyzed by the ΔΔCT method. Glyceraldehyde 3-phosphate dehydrogenase (*GADPH*) was used as the reference gene (a housekeeping gene). Prior to differential expression analyzes, efficiency of both *GADPH* and *RSK4* was determined using dilution series. The real-time qPCR procedure was performed according to the instruction of the kit used (Pars Tous, Iran), however, due to low *RSK4* expression levels in normal tissues and the possibility of expression reduction in our sample cells (based on our hypothesis), the annealing temperature was reduced, whilst the number of the cycles was increased (40 cycles of 30 seconds for denaturation at 95˚C, 40 seconds for annealC ing at 56˚C and 30 seconds for extension at 72˚C). Primers used are shown in Table 1. 

**Table 1:** Primers used for PCR


*RSK4*	F: 5′-TGCTCAAGGTTCTTGGTCAG-3′
	R: 5′-TTTGTCCGAACTCTGTCTCG-3′
*GAPDH*	F: 5′-TGCACCACCAACTGCTTAGC-3′
	R: 5′-GGCATGGACTGTGGTCATGAG-3′


PCR; Polymerase chain reaction.

### Statistical analysis

Data were analyzed using SPSS (Version 11.5). Initially, descriptive and then comparative analyses of parameters were performed. Due to the non-normal distribution of expression values, sample means were compared using Mann-Whitney test and correlation analysis was undertaken by Spearman’s test. P≤0.05 was considered to be significant.

## Results

After exclusion of five samples, 40 patients diagnosed with AML and 10 individuals as the control group were analyzed. In the patient group, 24 (60%) were male and 16 (40%) were female, while in the control group, 5 (50%) were male and 5 (50%) were female. The average age ± SD was 31 ± 17 and 29 ± 5.9 years in the patient and the con- trol groups respectively. The mean age difference between two groups was not significant (P=0.544). Differences between AML patients and the control group in red blood cell (RBC) and Platelet (PLT) count, Haematocrit (HCT) and Hemoglobin (Hb) were significant (P<0.05), however, white blood cell (WBC) count was not significantly different. Table 2 shows results of CBC in two groups.

Among the AML patients, the most common subtype (according to the FAB classification) was the AML-M3 (Table 3) and also the most common cytogenetic abnormality was t(15;17) (Table 4). The average expression level fold-change of *RSK4* in the patient group compared with the control group was 0.0041 ± 0.0048 (about 250-fold down-regulation) that shows a significant reduction (P<0.001). The expression level change was also analyzed in different morphologic subtypes. Among these, the M3 variant had the lowest and the M6 variant had the highest expression levels but these differences were not significant. Among the AML with chromosomal abnormalities, a significant decrease of *RSK4* expression was observed in AML with t(15;17) (P=0.004). We also analyzed correlation between the rates of *RSK4* expression in the AML group and their CBC parameters and observed only a correlation between RBC and gene expression rate (r=0.481, P<0.001).

**Table 2:** Results of complete blood count analysis in AML and control groups


Parameters	AML (Mean ± SD)	Control (Mean ± SD)	P value

RBC×10^6^/μl	2.94 ± 0.94	4.9 ± 0.59	<0.001
WBC×10^3^/μl	26.79 ± 33.3	7.19 ± 1.48	0.174
PLT×10^3^/μl	76.45 ± 74.72	204.9 ± 54.5	<0.001
HCT (%)	26.4 ± 8.35	42.1 ± 4.3	<0.001
Hb (g/dl)	8.46 ± 3.05	15.1 ± 1.6	<0.001


AML; Acute myeloid leukemia, RBC; Red blood cell, WBC; White blood cell, PLT; Platelet, HCT; Haematocrit and Hb; Hemoglobin.

**Table 3:** Frequencies of morphologic subtypes and rate of fold-change in the AML group


Morphologic subtype	n (%)	Fold change (Mean expression rate)

M0	1 (2.5%)	0.008
M1	4 (10%)	0.002
M2	6 (15%)	0.0034
M3	14 (35%)	0.0025
M3v	3 (7.5%)	0.00
M4	7 (17.5%)	0.0026
M5	4 (10%)	0.006
M6	1 (2.5%)	0.016


AML; Acute myeloid leukemia.

**Table 4:** Frequencies of cytogenetic abnormalities and rate of fold change in the AML group


Chromosomal disorder	n (%)	Fold change (Mean expression rate)

t(15;17)	10 (25%)	0.0004
t(8;21)	2 (5%)	0.0015
inv(16)	2 (5%)	0.0035
Other^*^	26 (65%)	0.0057


AML; Acute myeloid leukemia and ; Patients negative for the three chromosomal disorders mentioned.

## Discussion

Due to the high activity of ERK in some AML progenitors and ability of RSK to inhibit ERK, we hypothesized that *RSK4* may play a role in AML leukemogenesis. As a result, we analyzed possible expression changes of *RSK4* in AML. The expression level of *RSK4* decreased in AML patients and this decrease was significant in AML with the t(15;17) chromosomal disorder. According to some studies, it is possible that decrease in *RSK4* expression results in overactivating the ERK/MPAK signaling pathway in AML cells and finally myeloid proliferation (11). The cause of the huge reduction in *RSK4* expression (~250-fold down-regulation) may be either the low expression of the gene in normal tissues or down regulation of *RSK4* in AML as a tumor suppressor. The reason for reduction of *RSK4* expression may relate to the missense mutation in the N-terminal of *RSK4* kinase that has also been observed in primary malignant cells in lung cancer (22). AML is a malignancy with various patterns of genetic and epigenetic changes of which the latter, especially methylation, is responsible for leukemogenesis (24). The CpG island hyper methylation phenotype has been observed in endometrial cancer cells (25). This methylation in the endometrial cancer occurs on CpG islands of *RSK4*, thus resulting in a reduction in expression of *RSK4* (22). We therefore speculate that the methylation of the *RSK4* in AML may have resulted in decreasing the gene expression. Eisinger-Mathason et al. (26) showed that an increase in *c-MYC* expression results in an increase in the expression of *RSK4* in breasts cancer. They also suggested that *RSK4* is a putative tumor suppressor in breast cancer. Salvatori et al. (27) observed an increase in C-myc expression level in AMLs with mutation in *FLT3* and t(15;17). However, we observed decrease in expression of *RSK4* in AML with t(15;17). It is possible that this difference may be due to the double-sided effects of *c-MYC* on cellular growth and proliferation (28). In most studies, evaluation of *RSK4* expression has been performed on transgenic mice (26), fibroblast cell lines (IMR90) (3), colon carcinoma cell lines (HCT116) (29) and other similar cell lines. Since the expression rate of *RSK4* changes by stress (3), these studies were under more controlled circumstances to reduce the effect of such interferences, while our study was performed on recently diagnosed patients with no control on their circumstances which could have changed the expression level. Therefore, our results are closer to real-life situations and more applicable. AML with t(15;17) has a good prognosis (30), whereas reduction of *RSK4* expression as a tumor suppressor is associated with poor prognosis in some malignancies (22). However, various factors affect the prognosis of AML, especially the type of genetic abnormality (according to the WHO classification).

Other members of the RSK family also act as substrates for ERK and activate some transcription factors which play important roles in some malignancies and may be effective factors in leukemias (31). It was suggested that only *RSK4* has a tumor suppression role and other members have the same effect as that of ERK, however, in a recent study, it was shown that RSK3 also inhibits ovary cancer cell lines (32) and in 50% of ovarian tumor cells this gene is down-regulated (26).

## Conclusion

We observed down-regulation of *RSK4* (as a tumor suppressor) in AML patients. Given that the ERK/MPAK pathway exhibits increased activity in AML progenitors and the suppressive effect of *RSK4* on this pathway, it would be interesting to investigate the effect of *RSK4* on AML further.
